# Investigation of SARS-CoV-2 Transmission Associated With a Large Indoor Convention — New York City, November–December 2021

**DOI:** 10.15585/mmwr.mm7107a4

**Published:** 2022-02-18

**Authors:** Samira Sami, Libby Horter, Diana Valencia, Isabel Thomas, Mary Pomeroy, Brianna Walker, Sarah E. Smith-Jeffcoat, Jacqueline E. Tate, Hannah L. Kirking, Nang Thu Thu Kyaw, Rebecca Burns, Kathleen Blaney, Vajeera Dorabawila, Rebecca Hoen, Zachary Zirnhelt, Cody Schardin, Anna Uehara, Adam C. Retchless, Vance R. Brown, Yonathan Gebru, Charles Powell, Stephen M. Bart, Johanna Vostok, Hannah Lund, Jessica Kaess, Megan Gumke, Randy Propper, Deepam Thomas, Mojisola Ojo, Alison Green, Morgan Wieck, Erica Wilson, Ryan J. Hollingshead, Sheila V. Nunez, Dawn M. Saady, Charsey Cole Porse, Kyle Gardner, Daniel Drociuk, Julia Scott, Taidy Perez, Jim Collins, Julie Shaffner, Ian Pray, Laura T. Rust, Shane Brady, Janna L. Kerins, Richard A. Teran, Victoria Hughes, Victoria Sepcic, Eleanor W. Low, Sarah K. Kemble, Alexandra Berkley, Kate Cleavinger, Haytham Safi, Lindsey Martin Webb, Scott Hutton, Courtney Dewart, Kristen Dickerson, Eric Hawkins, Javeria Zafar, Anna Krueger, Dena Bushman, Bailee Ethridge, Katrina Hansen, Jake Tant, Christy Reed, Carla Boutwell, Jennifer Hanson, Meagan Gillespie, Matthew Donahue, Pilar Lane, Ruby Serrano, Lorena Hernandez, Michelle A. Dethloff, Ruth Lynfield, Kathryn Como-Sabetti, Emily Lutterloh, Joel Ackelsberg, Jessica N. Ricaldi

**Affiliations:** ^1^CDC COVID-19 Emergency Response Team; ^2^Goldbelt C6, LLC, Chesapeake, Virginia; ^3^Oak Ridge Institute for Science and Education, Oak Ridge, Tennessee; ^4^New York City Department of Health and Mental Hygiene, New York; ^5^New York State Department of Health; ^6^Minnesota Department of Health; ^7^Connecticut Department of Public Health; ^8^Epidemic Intelligence Service, CDC; ^9^Massachusetts Department of Public Health; ^10^Pennsylvania Department of Health; ^11^CSTE Applied Fellow, Council State and Territorial Epidemiologists, Atlanta, Georgia; ^12^Philadelphia Department of Public Health, Philadelphia, Pennsylvania; ^13^Division of Disease Control and Health Protection, Florida Department of Health; ^14^New Jersey Department of Health; ^15^Rhode Island Department of Health; ^16^North Carolina Department of Health and Human Services; ^17^Delaware Division of Public Health, Dover, Delaware; ^18^Virginia Department of Health; ^19^California Department of Public Health; ^20^South Carolina Department of Health & Environmental Control; ^21^Michigan Department of Health and Human Services; ^22^Tennessee Department of Health;^ 23^Division of State and Local Readiness, Office of Public Health Preparedness and Response, CDC; ^24^Wisconsin Department of Health Services; ^25^Arizona Department of Health Services; ^26^Chicago Department of Public Health, Chicago, Illinois; ^27^Nevada Department of Health and Human Services; ^28^Hawaii State Department of Health; ^29^Missouri Department of Health and Senior Services; ^30^Arkansas Department of Health; ^31^Colorado Department of Public Health and Environment; ^32^Idaho Department of Health and Welfare; ^33^Ohio Department of Health;^34^Indiana Department of Health; ^35^CDC Foundation, Atlanta, Georgia; ^36^Kentucky Department of Health; ^37^Maine Department of Health and Human Services; ^38^New Hampshire Division of Public Health Services; ^39^Utah Department of Health; ^40^West Virginia Department of Health & Human Resources; ^41^Mississippi State Department Health; ^42^State of Montana Department of Health and Human Services;^43^Nebraska Department of Health and Human Services; ^44^Puerto Rico Department of Health; ^45^North Dakota Department of Health.

During November 19–21, 2021, an indoor convention (event) in New York City (NYC), was attended by approximately 53,000 persons from 52 U.S. jurisdictions and 30 foreign countries. In-person registration for the event began on November 18, 2021. The venue was equipped with high efficiency particulate air (HEPA) filtration, and attendees were required to wear a mask indoors and have documented receipt of at least 1 dose of a COVID-19 vaccine.* On December 2, 2021, the Minnesota Department of Health reported the first case of community-acquired COVID-19 in the United States caused by the SARS-CoV-2 B.1.1.529 (Omicron) variant in a person who had attended the event (*1*). CDC collaborated with state and local health departments to assess event-associated COVID-19 cases and potential exposures among U.S.-based attendees using data from COVID-19 surveillance systems and an anonymous online attendee survey. Among 34,541 attendees with available contact information, surveillance data identified test results for 4,560, including 119 (2.6%) persons from 16 jurisdictions with positive SARS-CoV-2 test results. Most (4,041 [95.2%]), survey respondents reported always wearing a mask while indoors at the event. Compared with test-negative respondents, test-positive respondents were more likely to report attending bars, karaoke, or nightclubs, and eating or drinking indoors near others for at least 15 minutes. Among 4,560 attendees who received testing, evidence of widespread transmission during the event was not identified. Genomic sequencing of 20 specimens identified the SARS-CoV-2 B.1.617.2 (Delta) variant (AY.25 and AY.103 sublineages) in 15 (75%) cases, and the Omicron variant (BA.1 sublineage) in five (25%) cases. These findings reinforce the importance of implementing multiple, simultaneous prevention measures, such as ensuring up-to-date vaccination, mask use, physical distancing, and improved ventilation in limiting SARS-CoV-2 transmission, during large, indoor events.^† ^

An indoor convention in NYC with approximately 53,000 attendees was held during November 19–21, 2021. The facility was equipped with HEPA filters, and attendees were required to have documented receipt of at least 1 dose of COVID-19 vaccine and to use face masks while indoors. On December 2, 2021, the Minnesota Department of Health identified a case of COVID-19 caused by the Omicron variant in an attendee. State and local health departments collaborated with CDC to determine the extent of transmission during the convention and to make public health recommendations.

Two primary data sources were used in this investigation. The first was a list of attendees residing within the jurisdictions of participating state and local health departments. These attendees were matched with data from COVID-19 surveillance systems using personal identifiers (name and complete or partial address). Health departments identified positive and negative SARS-CoV-2 test results, demographic data, and vaccination histories^§^ for attendees. An event-associated case was defined as SARS-CoV-2 infection confirmed by reverse transcription–polymerase chain reaction or antigen testing in an event attendee during November 18–December 5, 2021. Sequencing of available specimens was conducted by state public health laboratories using multiple platforms^¶^; variant identification results were shared with CDC.

The second data source, an online anonymous survey, was administered via text message (29,766 text messages sent) and email (28,893 emails delivered) to approximately 35,000 attendees from 52 jurisdictions with available contact information, during December 11–19, 2021. Respondents were asked to report SARS-CoV-2 testing history and results, COVID-19 vaccination status, symptom history,** and exposure data during the event, and close contacts during and after the event. Available surveillance information and survey responses from U.S. resident attendees who received positive and negative test results were compared. Wilcoxon rank-sum tests were used for continuous data, and Pearson’s chi-square or Fisher’s exact tests were used for categorical data; statistical significance was defined as p<0.05.^††^ This activity was reviewed by CDC and was conducted consistent with applicable federal law and CDC policy.^§§^

Using COVID-19 surveillance systems, 48 public health jurisdictions reviewed data for 34,072 registered attendees; 39 jurisdictions reported a positive or negative result for 4,560 (13.4%) attendees, including 13 (<1%) self-tests^¶¶^ (from two states) (Table 1). Among 3,845 (84.3%) attendees with test and vaccination data, 3,248 (84.5%) had received a primary vaccination series, an additional 467 (12.1%) had received a booster dose,*** and 130 (3.4%) were partially vaccinated.

Among the 4,560 attendees with test result data, 119 (2.6%) event-associated cases were identified by January 6, 2022, from 16 jurisdictions (Figure). Among event-associated cases the median age was 26.5 years (IQR = 23.0–36.6 years), 65 (54.6%) were New York residents, and among 116 with gender data available, 54 (46.6%) were male (Table 1). Vaccination information was available for 88 persons with event-associated cases, 85 (96.6%) completed vaccination, including five who had received a booster dose. Among event-associated cases, the median interval from completing primary vaccination series to positive test result was 210 days (IQR = 193–232 days), and from booster dose to positive test result was 14 days (IQR = 12–20 days). Among the 3,630 (80%) test-negative attendees who completed primary vaccination or received booster dose, the median interval from completion of primary vaccination series to test date was 207 days (IQR = 187–225 days) and from receipt of booster dose to test date was 34 days (IQR = 22–66 days). One attendee with event-associated COVID-19 was hospitalized; no deaths were reported.

**FIGURE:**
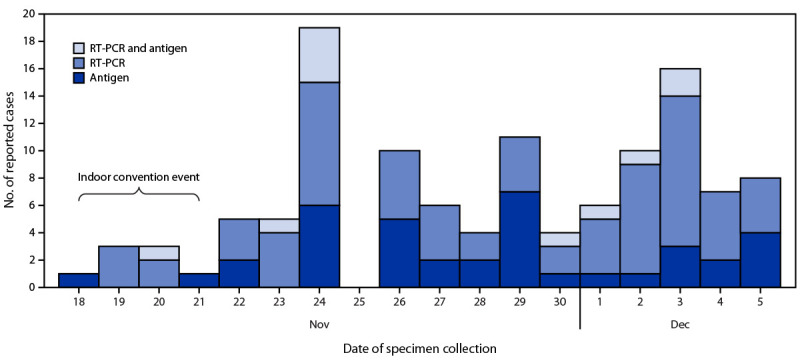
Event-associated cases* of SARS-CoV-2 infection (n = 119)^†^ among attendees of a large indoor convention in New York City, by date of specimen collection and test type^§^ — 16 jurisdictions, November–December 2021 **Abbreviation:** RT-PCR = reverse transcription–polymerase chain reaction. * Reported by health department COVID-19 surveillance systems. ^† ^Among 4,560 attendees with test result data, 119 (2.6%) event-associated cases were identified by January 6, 2022, from 16 jurisdictions. ^§^ Antigen, RT-PCR, and RT-PCR and antigen are mutually exclusive groups.

**TABLE 1:** Demographic characteristics and vaccination history of persons who attended a large, indoor convention in New York City and had a SARS-CoV-2 test result during November 18–December 5 reported by health department COVID-19 surveillance systems, by test result — 39 U.S. jurisdictions, November–December 2021

Characteristic	No. (%)			p-value*
	Total	Positive test	Negative test	
	(N = 4,560)	(n = 119)	(n = 4,441)	
**Demographic**				
**Sex**				
Known no.^†^	4,485 (98.4)	116 (97.5)	4,369 (98.4)	NA
Male	2,364 (52.7)	54 (46.6)	2,310 (52.9)	0.18
Female	2,121 (47.3)	62 (53.4)	2,059 (47.1)	
**Median age, yrs, (IQR)**	26.1 (22.2–31.4)	26.5 (23.0–36.6)	26.1 (22.2–31.4)	0.18
**State of residence**				
New York	3,967 (87.0)	65 (54.6)	3,902 (87.9)	<0.01
Outside of New York	593 (13.0)	54 (45.4)	539 (12.1)	
**Vaccination history**				
**Vaccination status**				
Known no.^†^	3,845 (84.3)	88 (73.9)	3,757 (84.6)	NA
Primary vaccination series received	3,248 (84.5)	80 (90.9)	3,168 (84.3)	0.17
Booster dose received	467 (12.1)	5 (5.7)	462 (12.3)	
Partially vaccinated	130 (3.4)	3 (3.4)	127 (3.4)	
**Days from booster dose to test date** ^§^				
Known no.^†^	467 (100)	5 (100)	462 (100)	NA
Median	34	14	34	<0.01
IQR	22–66	12–20	22–66	NA
**Days from primary vaccination series to test date** ^¶^				
Known no.^†^	3,248 (100)	80 (100)	3,168 (100)	NA
Median	207	210	207	0.34
IQR	188–225	193–232	187–225	NA

**Abbreviation:** NA = not applicable.

* Testing for statistical significance was conducted using nonparametric tests (i.e., Wilcoxon rank-sum) to compare continuous data and Pearson’s chi-square or Fisher's exact test to compare categorical data. Statistical significance was defined as p<0.05.

^†^ “Known no.” is defined as number of persons for individual variables that did not have missing data.

^§^ Percentage among those who received a booster dose.

^¶^ Percentage among those who received a primary vaccination series.

Genomic sequencing of 20 specimens identified the Delta variant (AY.25 and AY.103 sublineages) in 15 (75%) cases, and the Omicron variant (BA.1 sublineage) in five (25%). All attendees with Omicron cases were part of a known epidemiologic and phylogenetic cluster (*2*); no Delta variant cases were part of a cluster.^†††^

Among 7,259 respondents from the online survey (approximately 21% response rate) across 48 jurisdictions, 4,259 attendees reported receiving a COVID-19 test during November 18–December 5, 2021 (Table 2). Among these, 48 (1.1%) respondents from 10 jurisdictions reported SARS-CoV-2 infections during the investigation date range (including six from self-tests). The median age among test-positive attendees was 28 years (IQR = 23.0–35.0 years), 13 (27.7%) of 47 with reported gender were male, 15 (32.6%) of 46 with reported race/ethnicity were non-Hispanic White, and 19 (42.2%) of 45 reporting residency were New York residents. Among 47 test-positive survey respondents reporting vaccination information, 37 (78.7%) completed a primary vaccination series, six (12.8%) received a booster dose, and four (8.5%) were partially vaccinated. Among 4,157 test-negative respondents, 2,274 (54.7%) completed primary vaccination, 1,511 (36.3%) received a booster dose, and 372 (8.9%) were partially vaccinated. The median interval from booster dose receipt to a SARS-CoV-2–positive specimen was 12 days (IQR = 10–21 days) and to a negative specimen was 20 days (IQR = 10–35 days).

**TABLE 2:** Demographic characteristics, potential exposures, and close contacts of New York City convention attendees who participated in an anonymous online survey and self-reported SARS-CoV-2 test results during November 18–December 5, by test result — 48 U.S. jurisdictions, November–December 2021

Characteristic	No. (%) of respondents			p-value^†^
	Total receiving test*	Positive test	Negative test	
	(N = 4,259)	(n = 48)	(n = 4,211)	
**Demographic**				
**Gender**				
Known no.^§^	4,204 (98.7)	47 (97.9)	4,157 (98.7)	NA
Female	2,107 (50.1)	31 (66.0)	2,076 (49.9)	0.13
Male	1,834 (43.6)	13 (27.7)	1,821 (43.8)	
Transgender	144 (3.4)	1 (2.1)	143 (3.4)	
Other^¶^	119 (2.8)	2 (4.3)	117 (2.8)	
**Median age, yrs, (IQR)**	26 (22.0–31.0)	28 (23.0–35.0)	26 (22.0–31.0)	0.26
**Race/Ethnicity**				
Known no.^§^	4,175 (98.0)	46 (95.8)	4,129 (98.1)	NA
Asian, non-Hispanic	1,127 (27.0)	12 (26.1)	1,115 (27.0)	0.54
Black, non-Hispanic	421 (10.1)	3 (6.5)	418 (10.1)	
Hispanic, any race	1,097 (26.3)	10 (21.7)	1,087 (26.3)	
Other, non-Hispanic	311 (7.4)	6 (13.0)	305 (7.4)	
White, non-Hispanic	1,219 (29.2)	15 (32.6)	1,204 (29.2)	
**State of residence**				
Known no.^§^	4,111 (96.5)	45 (93.8)	4,066 (96.6)	NA
New York	2,540 (61.8)	19 (42.2)	2,521 (62.0)	<0.01
Outside of New York	1,571 (38.2)	26 (57.8)	1,545 (38.0)	
**Vaccination history**				
**Vaccination status**				
Known no.^§^	4,204 (98.7)	47 (97.9)	4,157 (98.7)	NA
Primary vaccination series received	2,311 (55.0)	37 (78.7)	2,274 (54.7)	<0.01
Booster dose received	1,517 (36.1)	6 (12.8)	1,511 (36.3)	
Partially vaccinated	376 (8.9)	4 (8.5)	372 (8.9)	
**Days from booster dose to test date**				
Known no.^§^ (% of those who received booster)	838 (55.2)	6 (100)	832 (55.1)	NA
Median	20	12	20	0.58
IQR	10–34	10–21	10–35	NA
**Prevention measures and exposures**				
**Mask use over nose and mouth while indoors**				
Known no.^§^	4,246 (99.7)	48 (100)	4,198 (99.7)	NA
Always	4,041 (95.2)	46 (95.8)	3,995 (95.2)	0.07
Sometimes	184 (4.3)	1 (2.1)	183 (4.4)	
Never	11 (0.3)	1 (2.1)	10 (0.2)	
Rarely	10 (0.2)	0 (—)	10 (0.2)	
**Type of mask used**				
Known no.^§^	4,230 (99.3)	47 (97.9)	4,183 (99.3)	NA
Surgical	1,872 (44.3)	21 (44.7)	1,851 (44.3)	0.89
Cloth	1,812 (42.8)	21 (44.7)	1,791 (42.8)	
N94/N95	546 (12.9)	5 (10.6)	541 (12.9)	
**Outside activities during convention attendance**				
Known no.^§^	4,259 (100)	48 (100)	4,211 (100)	NA
Outdoor sightseeing	798 (18.7)	10 (20.8)	788 (18.7)	0.71
Indoor sightseeing	544 (12.8)	9 (18.8)	535 (12.7)	0.20
Bars	297 (7.0)	8 (16.7)	289 (6.9)	0.02
Nightclubs	130 (3.1)	5 (10.4)	125 (3.0)	0.01
Karaoke	112 (2.6)	9 (18.8)	103 (2.4)	<0.01
**Ate or drank indoors in close proximity to others for at least15 minutes**				
Known no.^§^	4,237 (99.5)	48 (100)	4,189 (99.5)	NA
Yes	1,859 (43.9)	30 (62.5)	1,829 (43.7)	0.01
**Shared a room with another person during convention attendance**				
Known no.	4,171 (97.9)	45 (93.8)	4,126 (98.0)	NA
Yes	1,440 (34.5)	21 (46.7)	1,419 (34.4)	0.11
**Travel to other states in the 14 days before or after travel to NYC (non-New York residents)**				
Known no.^§^	4,200 (98.6)	47 (97.9)	4,153 (98.6)	NA
Yes	679 (16.2)	10 (21.3)	669 (16.1)	0.32
**Travel outside of the United States in the 14 days before or after travel to NYC (non-New York residents)**				
Known no.^§^	4,189 (98.4)	47 (97.9)	4,142 (98.4)	NA
Yes	45 (1.1)	0 (—)	45 (1.1)	1.00
**Contact with anyone who traveled outside the United States (14 days before positive test or symptoms)**				
Known no.^§^	3,977 (93.4)	47 (97.9)	3,930 (93.3)	NA
Yes	25 (0.6)	1 (2.1)	24 (0.6)	0.26
**Exposure notification and close contacts**				
**Used the COVID Alert NYC app during the event** ^**^				
Known no.^§^	4,249 (99.8)	47 (97.9)	4,202 (99.8)	NA
Yes	271 (6.4)	4 (8.5)	267 (6.4)	0.54
New York resident	209 (80.1)	1 (33.3)	208 (80.6)	0.10
Non-New York resident	52 (19.9)	2 (66.7)	50 (19.4)	
No	3,978 (93.6)	43 (91.5)	3,935 (93.6)	
**Received exposure notification via COVID Alert NYC app during or after the event**				
Known no.^§^ (% among those who used app)	270 (99.6)	4 (100)	266 (99.6)	NA
Yes	75 (27.8)	1 (25)	74 (27.8)	1.0
No	195 (72.2)	3 (75)	192 (72.2)	
**Had a close contact report a positive COVID-19 test result within 10 days of respondent’s symptom onset or test result**				
Known no.^§^	335 (7.9)	34 (70.8)	301 (7.1)	NA
Yes	33 (9.9)	15 (44.1)	18 (6.0)	<0.01
**Met, interacted, or worked with at least one person during the convention who reported a positive SARS-CoV-2 test result since attending the convention**				
Known no.^§^	4,245 (99.7)	48 (100)	4,197 (99.7)	NA
Yes	87 (2.0)	7 (14.6)	80 (1.9)	<0.01

**Abbreviations:** NA = not applicable; NYC = New York City.

* Self-reported SARS-CoV-2 test results from an online, anonymous survey.

^†^ Testing for statistical significance was conducted using nonparametric tests (i.e., Wilcoxon rank-sum) to compare continuous data and Pearson’s chi-square or Fisher's exact test to compare categorical data. Statistical significance was defined as p<0.05.

^§^ Known no. is defined as number of persons for individual variables that did not have missing data.

^¶^ Other is defined in the survey as “none of these.”

** COVID Alert NYC app is a voluntary, anonymous, exposure-notification smartphone application that provides an alert if a person was in close contact with someone who receives a positive SARS-CoV-2 test result. The period for exposure notification was November 18–December 5, 2021.

Among the 48 test-positive respondents, 34 (70.8%) reported COVID-19 compatible symptoms, compared with 312 (7.4%) of 4,203 test-negative respondents. Nasal congestion or runny nose (91.2%) and fatigue (88.2%) were common symptoms reported among test-positive respondents; among test-negative respondents, nasal congestion or runny nose (223 of 310; 71.9%) and sore throat (191 of 305; 62.6%) were most commonly reported. No hospitalizations were reported.

Test-positive survey respondents reported engaging in certain activities more frequently than did test-negative respondents, including attending bars (16.7% versus 6.9%), karaoke (18.8% versus 2.4%), or nightclubs (10.4% versus 3.0%) outside of the convention, and eating or drinking indoors near others for at least 15 minutes at the convention (62.5% versus 43.7%) (all p<0.05). Differences were also found in reporting close contact with someone with a positive COVID-19 test result within 10 days of symptom onset or test result (44.1% versus 6.0%) (p<0.05). Most (4,041 [95.2%]) attendees, reported always wearing a mask over their nose and mouth while indoors; no difference was found in type of mask used by test result. Among 4,245 survey respondents, 87 (2.0%) reported knowing at least one person (mean = 2.4) whom they met, interacted with, or worked with during the event who received a positive SARS-CoV-2 test result since attending the event.

On December 2, 2021, after identification of the first Omicron case, CDC issued an Epidemic Information Exchange (Epi-X) notification to U.S. health departments to identify COVID-19 cases among event attendees. On December 3, 2021, the NYC Test and Trace program^§§§^ alerted registered attendees via text and email messages to get tested immediately, wear a face mask, and maintain physical distance from others.

## Discussion

This investigation identified 119 event-associated COVID-19 cases, including one hospitalization. A parallel epidemiologic investigation describing a cluster of attendees with social links (*2*) revealed that at least seven U.S.-based persons potentially attended the event during their infectious period.^¶¶¶^ Despite these potential exposures and multiple introductions as evidenced by genomic identification of at least three different SARS-CoV-2 variants and sublineages, findings from surveillance and survey data from a portion of attendees suggest that this large event did not lead to widespread transmission; 7-day average percentage of positive test results in NYC on December 5, 2021, (3.0%) was similar to that in this investigation (2.6%) *(**3**)*. Omicron variant accounted for <5% of sequenced cases in NYC by December 4, 2021; transmission could have been higher had the convention occurred after Omicron became the dominant variant *(**4**)*.

Reported prevention measures (vaccination requirements, enforcement of mask use, and avoidance of unmasked indoor settings), and a venue with HEPA filtration likely accounted for the limited number of event-associated cases. Indoor gatherings in which prevention measures do not occur have been shown to increase the spread of COVID-19 (*5*–*8*). In addition, transmission to household contacts, including to vaccinated or previously infected persons, was documented in the related cluster investigation and a previous Omicron investigation (*2*,*9*).

The findings in this report are subject to at least six limitations. First, case finding and survey distribution were limited to a registration list of 35,613 ticket purchasers, but the event organizer reported that approximately 53,000 persons had attended. Second, matching attendees with case surveillance data was conducted by jurisdictions using only name and address, which potentially limited the number of cases and vaccination records identified or misidentified attendees. In addition, self-testing results were not included by most jurisdictions. Third, few specimens were available for sequencing (17% of event-associated cases). Fourth, the limited reach (14% of reported attendees) and low response rate of the survey (approximately 21%) can increase potential biases if respondents differ systematically from nonrespondents. Fifth, responses were subject to self-reporting bias; attendees who sought testing might be more likely to respond or respond according to social desirability bias. Finally, the definition of event-association case could have included cases from transmission unrelated to the event.

Findings from this survey and a related cluster investigation (*2*) of a portion of attendees suggest transmission occurred primarily among social circles and during indoor unmasked activities during the event rather than at official event activities. These findings reinforce the importance of implementing multiple, simultaneous prevention measures, such as ensuring up-to-date vaccination, mask use, physical distancing, and improved ventilation in limiting SARS-CoV-2 transmission, including highly transmissible Delta and Omicron variants, during large indoor events.

**Summary**

**What is already known about this topic?**

The SARS-CoV-2 Delta (B.1.617.2) and Omicron (B.1.1.529) variants are highly transmissible. Outbreaks have been reported among vaccinated populations in indoor settings where mask use was limited.

**What is added by this report?**

Despite multiple introductions as evidenced by detection of at least three sublineages of SARS-CoV-2, this investigation did not find evidence of widespread transmission among a highly vaccinated population at a large event in an indoor setting where mask use was required and monitored.

**What are the implications for public health practice?**

Implementing multiple prevention measures (vaccinations and boosters, consistent mask wearing, enhanced indoor ventilation, and testing after text notification) can limit the transmission of SARS-CoV-2 at large events, including highly transmissible variants.

## Footnotes

* https://www1.nyc.gov/assets/home/downloads/pdf/executive-orders/2021/eeo-225.pdf

†
https://www.cdc.gov/coronavirus/2019-ncov/daily-life-coping/index.html

§ Persons who had received a primary vaccination series had completed all recommended doses of a Food and Drug Administration–authorized COVID-19 vaccine (2 doses of BNT162b2 [Pfizer-BioNTech] or mRNA-1273 [Moderna], or 1 dose of Ad.26.COV2.S (Janssen [Johnson & Johnson]) ≥14 days before specimen collection and had documentation in their state immunization information system or self-report of vaccination details (including vaccine product and dates of receipt). Persons who had received a booster dose had completed a primary vaccination series and received another dose of vaccination regardless of the time frame. Persons who had received only 1 dose of a 2-dose vaccination series or had completed vaccination <14 days before specimen collection were considered partially vaccinated.

¶ Sequencing platforms included Nanopore (Oxford Nanopore Technologies); NextSeq (Illumina); NovaSeq 6000 (Illumina); Miseq System (Illumina); PacBio Sequel II Systems (PacBio); and GridION (Oxford Nanopore Technologies).

** COVID-19–like symptoms were based on the Council of State and Territorial Epidemiologists surveillance case definition for COVID-19. https://ndc.services.cdc.gov/case-definitions/coronavirus-disease-2019-2020-08-05/

†† Respondents who did not confirm attending the convention or who resided outside the United States were excluded from the analysis.

§§ 45 C.F.R. part 46.102(l)(2); 21 C.F.R. part 56; 42 U.S.C. Sect. 241(d); 5 U.S.C. Sect. 552a; 44 U.S.C. Sect. 3501 et seq.

¶¶
https://www.cdc.gov/coronavirus/2019-ncov/testing/self-testing.html

***
https://www.cdc.gov/coronavirus/2019-ncov/vaccines/booster-shot.html

††† The closely linked Omicron cases involved lineage BA.1 SARS-CoV-2 with no discernable difference in the genomic regions with reliable sequence, consistent with the epidemiologic links.

§§§
https://www.nychealthandhospitals.org/test-and-trace/

¶¶¶ Infectious period for the cluster investigations was defined as 2 days before and 10 days after their symptom onset date.
